# Supplementary material for: Lung Extracellular Matrix Hydrogels-Derived Vesicles Contribute to Epithelial Lung Repair
Source: Polymers (Basel). 2022 Nov 14;14(22):4907. doi: 10.3390/polym14224907 (PMC9692679; doi:10.3390/polym14224907)
Supplement: Supplementary file 1 [file polymers-14-04907-s001.zip › polymers-2013024-supplementary.pdf]

Supplementary material

Lung Extracellular Matrix Hydrogels-Derived Vesicles Contribute to Epithelial Lung Repair

Anna Ulldemolins [1], Alicia Jurado [1], Carolina Herranz-Diez [1], Núria Gavara [1,2], Jorge Otero [1,2,3], Ramon Farré [1,3,4] and Isaac Almendros [1,3,4,*]

1 Unitat de Biofísica i Bioenginyeria, Facultat de Medicina i Ciències de la Salut, Universitat de Barcelona, 08036 Barcelona, Spain
2 The Institute for Bioengineering of Catalonia (IBEC), The Barcelona Institute of Science and Technology (BIST), 08028 Barcelona, Spain
3 CIBER de Enfermedades Respiratorias, 28029 Madrid, Spain
4 Institut d'Investigacions Biomèdiques August Pi i Sunyer, 08036 Barcelona, Spain
* Correspondence: isaac.almendros@ub.edu

Condition	Protein amount (µg/ml)
MSCs	6.72 ± 4.02
L-HG	15.43 ± 4.05
L-HG + MSCs	18.13 ± 3.58
L-HG NW	38.84 ± 4.34

Supplementary Table S1: Total protein added in each condition.

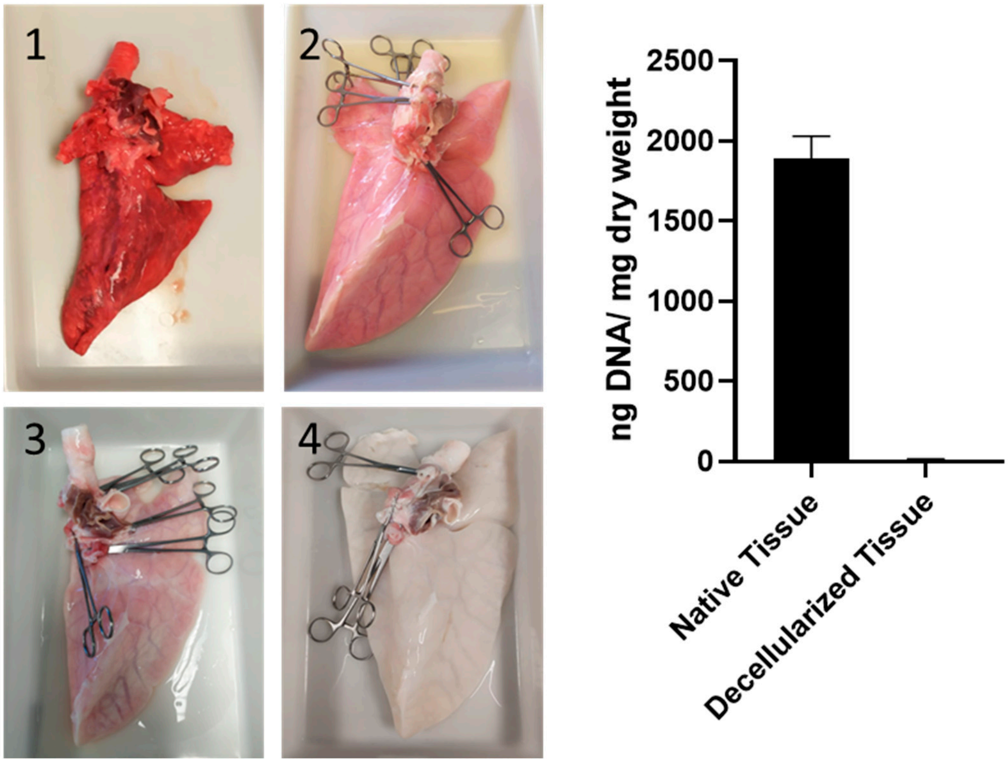

Supplementary Figure S1. Lung Decellularization. Left: Successive images of the porcine right lung through all the decellularization protocol (1: native, 4: decellularized). Right: DNA quantification on the lung tissue.

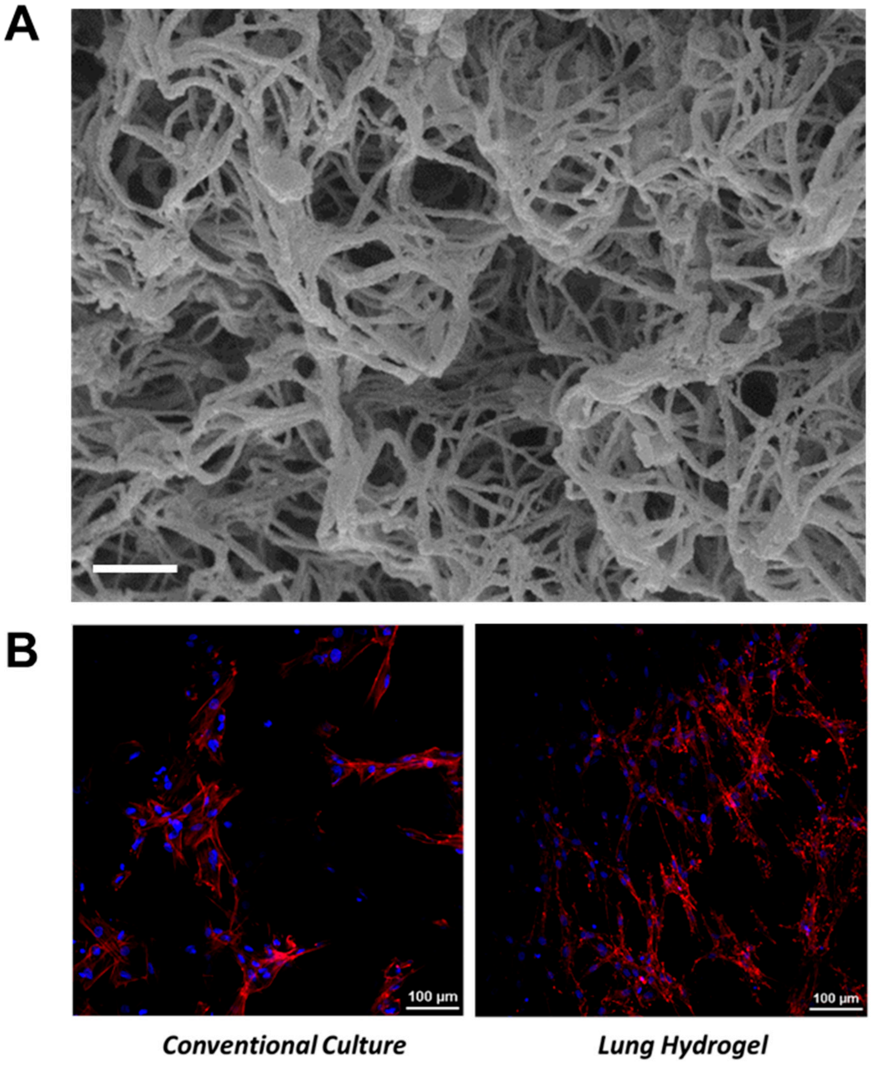

Supplementary Figure S2. (A) Scanning electron microscope (SEM) images of L-HG structure. Scale bar = 1 µm. (B) Cell Distribution and morphology of MSCs on the conventional cell culture and on L-HG. Actin staining (red) and nucleus (blue).
